# All-in-One Optofluidic Chip for Molecular Biosensing Assays

**DOI:** 10.3390/bios12070501

**Published:** 2022-07-09

**Authors:** Tyler Sano, Han Zhang, Ravipa Losakul, Holger Schmidt

**Affiliations:** Department of Electrical and Computer Engineering, University of California Santa Cruz, 1156 High Street, Santa Cruz, CA 95064, USA; tsano@ucsc.edu (T.S.); hzhan289@ucsc.edu (H.Z.); rlosakul@ucsc.edu (R.L.)

**Keywords:** optofluidics, biosensor, lab-on-chip, PDMS

## Abstract

Integrated biosensor platforms have become subjects of high interest for consolidated assay preparation and analysis to reduce sample-to-answer response times. By compactly combining as many biosensor processes and functions as possible into a single lab-on-chip device, all-in-one point-of-care devices can aid in the accessibility and speed of deployment due to their compact size and portability. Biomarker assay preparation and sensing are functionalities that are often carried out on separate devices, thus increasing opportunity of contamination, loss of sample volume, and other forms of error. Here, we demonstrate a complete lab-on-chip system combining sample preparation, on-chip optofluidic dye laser, and optical detection. We first show the integration of an on-chip distributed feedback dye laser for alignment-free optical excitation of particles moving through a fluidic channel. This capability is demonstrated by using Rhodamine 6G as the gain medium to excite single fluorescent microspheres at 575 nm. Next, we present an optofluidic PDMS platform combining a microvalve network (automaton) for sample preparation of nanoliter volumes, on-chip distributed feedback dye laser for target excitation, and optical detection. We conduct concurrent capture and fluorescence tagging of Zika virus nucleic acid on magnetic beads in 30 min. Target-carrying beads are then optically excited using the on-chip laser as they flow through an analysis channel, followed by highly specific fluorescence detection. This demonstration of a complete all-in-one biosensor is a tangible step in the development of a rapid, point-of-care device that can assist in limiting the severity of future outbreaks.

## 1. Introduction

Development of point-of-care lab-on-chip devices are of pressing demand as a result of recent vial outbreaks such as SARS-CoV-2 and Zika [1,2]. By increasing supply and portability of highly selective and sensitive diagnostic tools, diagnoses can be made significantly more accessible to the individual [3]. Several types of biosensors can be used to optically or electrically detect specific biomarkers that can be useful for effectively achieving this [4,5,6]. Optofluidic platforms can advance the level of integration by providing the ability to manipulate small volumes of fluids while incorporating the configurable functionality of photonic components and waveguiding capabilities into a single planar structure [7,8,9,10,11,12]. By developing lab-on-chip biosensors in polydimethylsiloxane (PDMS), we can exploit several advantages, including the ability for rapid prototyping, biocompatibility, mechanical elasticity, incorporation of microvalve-based sample processing, and optical transparency [13,14,15]. Development of optofluidic biosensors has dramatically increased over the last two decades, primarily being implemented on silicon based and PDMS platforms [16,17]. Integration of sensor functionalities has typically come in the form of hybrid structures that use different materials for separate functions of the device [18,19]. Here, we demonstrate a device where all components of the sensor (laser, sample preparation, and optical detection) are integrated into a single, “all-in-one” device fabricated solely of PDMS. By incorporating as many sensor functionalities on a single device as possible, errors involving sample transportation, contamination, and consistent optical excitation alignment can be avoided. Optofluidic biosensors have been demonstrated using varying detection methods that rely on evanescent fields such as ring resonator [20] and interferometer-based [18] detection. Another well-developed concept is the use of intersecting solid-core and liquid-core optical waveguides to produce a small excitation volume capable of achieving single particle detection sensitivity [15,21]. One disadvantage of using direct fiber-to-chip facet optical coupling is that alignment can be tedious and inconsistent. One solution to this is to excite the analyte using an out-of-plane external source, such as reported in [22] and [23], which is coupled using an external halogen lamp and laser diode, respectively, to the detection region from the out-of-plane direction. We have elected to integrate an optofluidic laser that is directly coupled to the analyte channel on-chip to further reduce inconsistencies in alignment. By integrating a laser on-chip, optical excitation can be directly coupled in plane to an orthogonal analyte channel via optical waveguides, and using dyes as gain media provides dynamic reconfigurability of the on-chip light source [24]. Various laser geometries have been implemented for on-chip optofluidic applications including droplet arrays, ring resonators, and Fabry–Perot resonators [25,26,27]. Distributed feedback (DFB) lasers are particularly useful in achieving low threshold pump powers and single mode lasing in on-chip systems. Here, the effective mode index of a waveguide mode is modulated with a spatial period that leads to efficient reflection at the desired lasing wavelength [28,29]. By incorporating the laser on our device, we can avoid the difficult and inconsistent alignment that is associated with aligning an external laser to a fluidic analysis channel while providing a reconfigurable and versatile optical excitation source to complete an all-in-one lab-on-chip device. The integration of sample preparation with optical detection was demonstrated previously by our lab with the detection of Zika nucleic acids and proteins [15]. Sample preparation was conducted by a series of pneumatically liftable valves inspired by [30] used to construct a magnetic bead-based assay, while optical excitation was carried out by an external laser diode coupled an orthogonal PDMS waveguide that intersects the fluidic analyte channel. Fluorescent signals were collected by a wider collection waveguide and coupled to an APD to construct a time domain trace. The inclusion of a fully integrated sample preparation stage that is capable of carrying out complex, multistep bioassays further set this device apart from other lab-on-chip devices such as [22] that relied on a single channel to uniformly mix two fluids together.

Here, we present an all-in-one system combining the three elements of sample preparation, laser, and optical detection in a complete, lab-on-chip biosensor. We first demonstrate the integration of the on-chip laser through optical detection of fluorescent microspheres at femtomolar concentration. We then demonstrate the complete all-in-one device by conducting both nonspecific and specific bioassay detection of 1 kbp dsDNA and Zika nucleic acids.

## 2. Materials and Methods

### 2.1. Device Design

**Figure 1:**
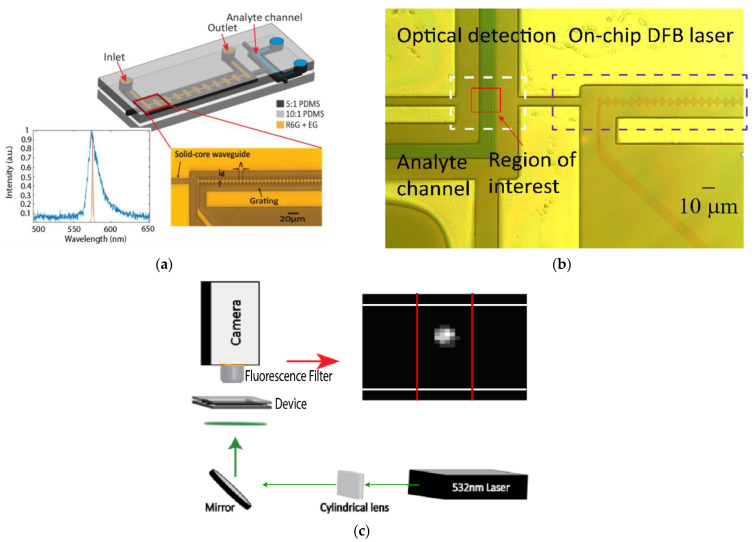
(**a**) PDMS device consisting of on-chip DFB laser integrated with an orthogonal analyte channel via a solid-core waveguide. Inset spectra illustrate lasing (orange) and amplified spontaneous emission (ASE) (blue) output from DFB laser when pumped above and below threshold, respectively. The lasing wavelength occurs at 575 nm. Microscope image shows DFB grating. (**b**) Microscope image of DFB grating filled with red food dye and intersection with analyte channel filled with blue food dye. (**c**) Experimental setup demonstrating optimal external pumping of DFB dye laser using an elliptically shaped 532 nm laser and camera collection of fluorescent signals through a 594 nm long pass fluorescence filter that is placed immediately before the camera. Camera image shows an example of a fluorescent microsphere imaged by the camera sensor. White lines indicate the bounds of the liquid-core analyte channel. Red lines indicate the bounds of the excitation waveguide.

Two separate device designs were used in this study. In the first device, a DFB laser with a sidewall grating is coupled to a fluidic analyte channel (Figure 1a). The m = 39 order DFB grating has an 8 µm period with 2.5 µm corrugations and was filled with rhodamine 6G that was dissolved in a solution of 85% ethylene glycol and 15% water at a concentration of 5 mM. The refractive index of this solution is 1.4174. Details of the operational principle and optical performance of this tunable on-chip laser can be found in [31]. A pulsed 532 nm Nd:YAG microchip laser (TEEM Photonics STG-03E-140, Meylan, France) at a repetition rate of 1–4 kHz was used to pump the DFB grating while emission occurred at a central wavelength of 575.2 nm. In sensing experiments, the laser was operated at 4 kHz, corresponding to a pump power of 12 mW. The emission from the on-chip DFB laser is coupled orthogonally into a fluidic analyte channel via a solid-core waveguide (Figure 1b).

**Figure 2:**
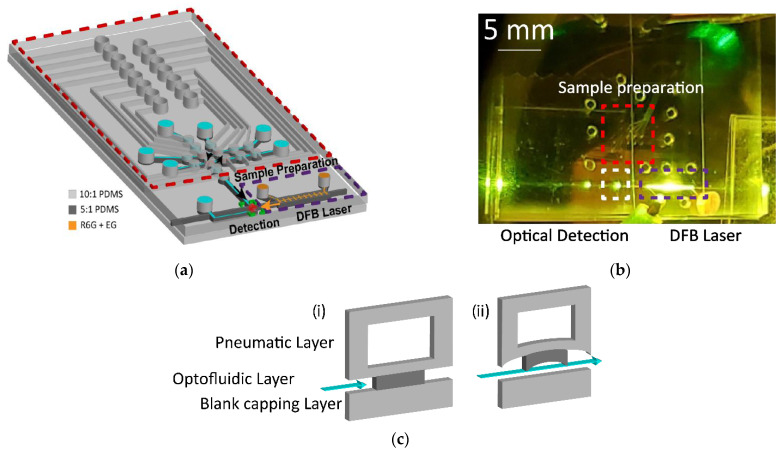
(**a**) PDMS device consisting of three distinct functional regions: sample preparation (red), light source (purple), and detection (green). (**b**) Top-down camera image of all-in-one chip. Scattering of DFB emission is visible through a long-pass filter that blocks pump laser. (**c**) Cross sectional diagrams of pneumatic lifting-gate valve structures with positive pressure used to close valves (i) and negative pressure used to lift valves and allows fluid flow (ii).

In the second design, sample preparation, on-chip laser, and optical detection are all interfaced into a single platform (Figure 2a). Fluid is transported throughout the device using a series of 15 pneumatically controlled lifting-gate valves that allow fluidic motion between liquid channels [30]. When negative pressure is applied to the valve, the PDMS valve lifts and allows fluid to move between adjacent valves (Figure 2c). Positive pressure is used to close valves and aid in pushing fluid in the direction of open valves. Valve structures measured 500 µm in diameter with the channel height being approximately 5–7 µm throughout the device. A custom-made control box implementing a LabView code allows for programs to carry out actuation of individual valves. The control box has input ports for vacuum and positive pressure and uses a National Instruments digital I/O board to control a series of 15 solenoid valves. The solenoid valves are then connected 15 individual pneumatic lines that are inserted into the pneumatic ports in the chip. The three distinct regions of the chip are indicated integration of sample preparation, on-chip laser, and optical detection site in Figure 2b.

### 2.2. Device Fabrication

**Figure 3:**
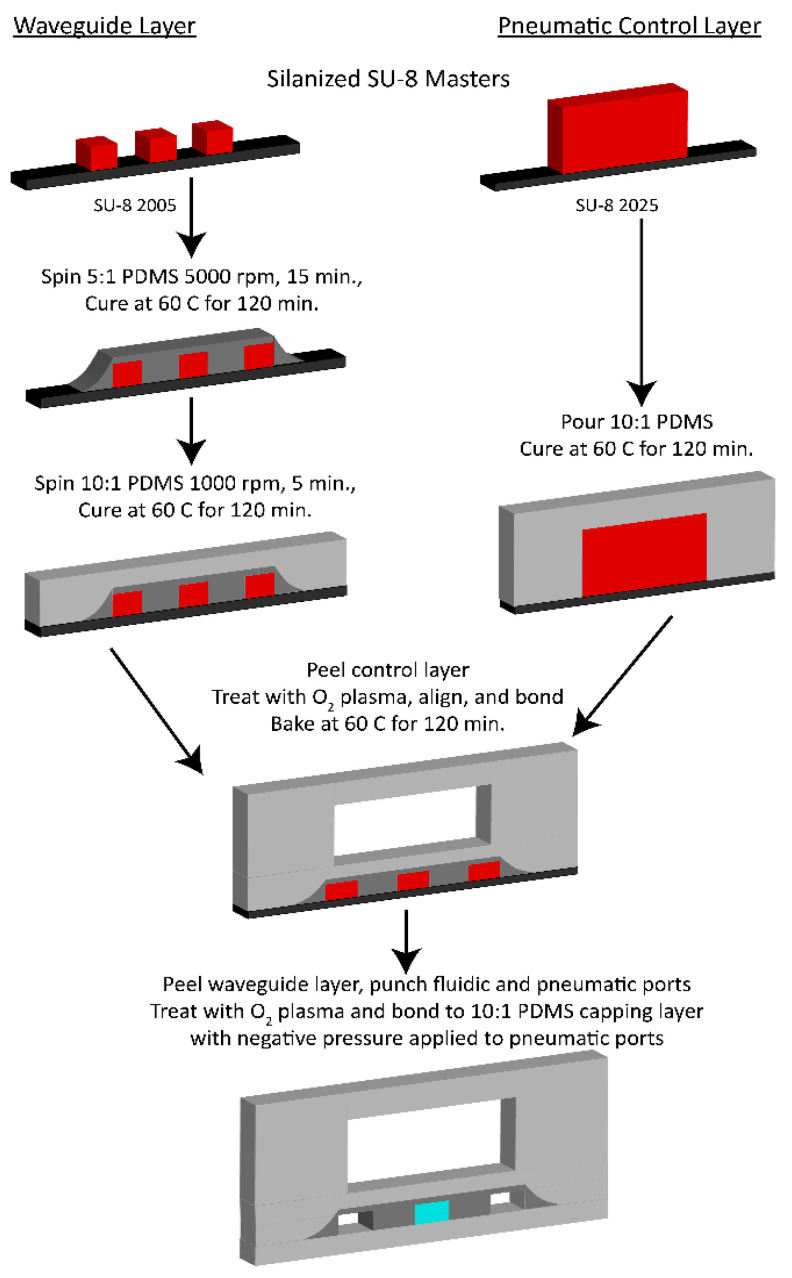
Fabrication of PDMS devices. A fabrication workflow is illustrated with 5:1 PDMS in dark gray, 10:1 PDMS in light gray, SU-8 in red, and silicon in black.

The all-in-one optofluidic device was fabricated in three layers using a soft lithography process outlined in [32] (Figure 3). One silicon master was fabricated using negative photoresist SU-8 2005 (Microchem, Round Rock, TX, USA) and consisted of fluidic channels and optical waveguides. A second master was fabricated using SU-8 2025 to produce taller features and contained pneumatic valves. Optical waveguides are created in the waveguide layer via total internal refraction where a refractive index difference is generated by using two different base-to-curing agent ratios when mixing PDMS (Sylgard 184) [33]. A 5:1 PDMS mixture that forms the waveguide core (refractive index 1.4170) is spun onto the waveguide master at 5000 RPM and for 15 min and then cured in a 60 °C oven for 2 h. A 10:1 mixture for the waveguide cladding (refractive index 1.4138) is then spun on at 1000 RPM for 5 min and then cured overnight. Simultaneously, a 10:1 mixture of PDMS was poured over both the pneumatic valve master as well as a blank wafer that was used as a capping layer. Both layers were then placed in the 60 °C oven to cure overnight.

After curing, the pneumatic layer was peeled from the master and treated with oxygen plasma along with the waveguide layer to activate the two surfaces. The layers were then aligned using a mask aligner (OAI instruments, Milpitas, CA, USA) such that the pneumatic valves were placed directly over the relevant fluidic channels, and then brought into contact. After curing for an additional 2 h to strengthen the bonding between the two layers, the waveguide layer was peeled from its silicon master and 1 mm punches were used to create liquid and pneumatic ports. Lastly, the exposed waveguide layer was then activated with oxygen plasma with the blank PDMS layer to cap the open channels and complete the three-layer device. During the final bonding, negative pressure was applied to the device to keep the pneumatic valves in the lifted state to prevent localized bonding.

### 2.3. Experimental Setup

The optofluidic devices were placed on a two glass slides to stabilize the device while maintaining an obstruction-free path for pumping of the DFB grating from the below. The DFB laser was pumped using bottom-up excitation via an elliptically shaped 532 nm beam using a cylindrical lens and a 45° mirror (Figure 1c). A camera mounted vertically above the chip was used to align the pump beam to the grating as well as monitor the fluidic motion throughout the chip and collect fluorescence signals in the analyte channel. A 594 nm long pass fluorescence filter is placed in the camera path to filter out laser emission. When collecting data, the collection window of the camera is reduced to a 40 × 40 pixel window. This allows for a framerate of 2000 frames per second, corresponding to a time resolution of 0.5 ms. This time resolution is short enough to capture individual fluorescence events in the detection area. The region of interest was then integrated frame-by-frame using ImageJ and plotted over time, yielding a time domain fluorescence trace.

### 2.4. Fluorescent Bead Detection

Fluorescent bead detection was used to investigate the integration of the DFB laser with the analyte channel as an optical excitation source (see Section 3.1). 1 µm red fluorescent beads (FluoSphere, Invitrogen, Waltham, MA, USA) were used as the analyte with a peak excitation of 580 nm and peak emission of 605 nm.

### 2.5. All-in-One Bioassay Detection

On-chip sample preparation and nonspecific detection capability of our device is shown through the optical detection of fluorescently stained nucleic acids. The target 1 kbp biotinylated dsDNA attached to a 1 µm streptavidin-coated magnetic bead (New England Biolabs, Ipswich, MA, USA). SYTO 64 intercalating dye was used as the fluorescent stain with a peak excitation of 599 nm and peak emission of 619 nm.

Zika nucleic acid detection was used to illustrate a specific binding bioassay on our device. A streptavidin-coated 1 µm magnetic bead served as the base of the assay. A biotinylated 50-base pair long ssDNA oligomer that is reverse complementary to the bottom half of the target sequence (KU321639.1, nt. 130–179) was used as the capture probe. The streptavidin coated magnetic bead and capture probe are the only portion of the assay that was hybridized off-chip. The completed bead construct was completed using standard protocol and washed three times to remove unconjugated capture probes. The target consisted of a 100-base pair long ssDNA oligomer (KU321639.1, nt. 121–220) that corresponds to the capsid region of the Zika genome. A 25-base pair long oligonucleotide (KU321639.1, nt. 187–211) that had an attached AlexaFluor 594 fluorophore that is the reverse complement of the top 25 bases of the target sequence serves as the target-specific probe. The reverse complementary capture sequence and reporter probe ensure specificity of the assay. Zika target, pull down, and fluorescent probes were purchased from IDT (Coralville, IA, USA).

## 3. Results

### 3.1. Fluorescent Bead Detection

The first goal was to demonstrate the integration of an on-chip laser with optical detection capabilities. To determine this, we optically excited fluorescent microspheres with our on-chip DFB laser and collected a time-resolved trace of the fluorescence signals.

After aligning the pump beam to the DFB channel, the grating was filled with the aforementioned R6G gain medium. Then, 3 µL of 1 µm red fluorescent beads at 4 × 10^7^ beads/mL concentration were placed on top of the analyte channel inlet and pulled through the channel using a constant negative pressure applied at the outlet. A 594 nm long pass fluorescence filter was placed in the camera path and a 2 min trace was taken at the intersection of the DFB emission and analyte channel to capture fluorescence signals.

**Figure 4:**
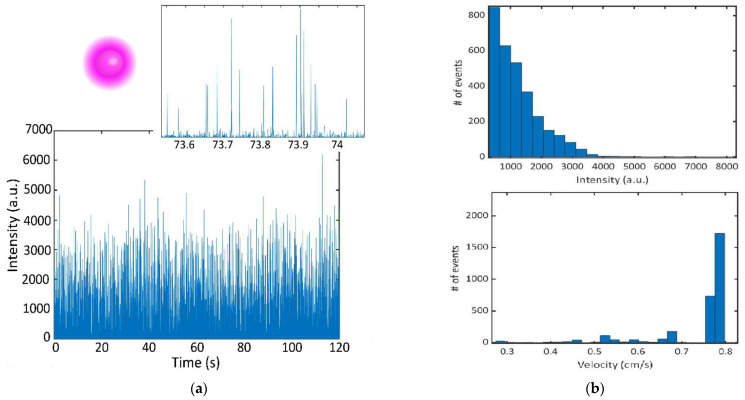
(**a**) Fluorescence time domain trace illustrating peaks observed from 4 × 10^7^ beads/mL 1 um fluorescent microspheres with an absorption maximum at 580 nm and emission maximum at 605 nm. Inset image shows a zoomed in window to illustrate individual events. (**b**) Intensity histogram of detected fluorescent events (top). The average intensity of events is 1222 with a standard deviation of 894. Velocity histogram of detected events (bottom). The average velocity of beads is 0.74 cm/s with a standard deviation of 0.01 cm/s.

A time domain fluorescence trace is illustrated in Figure 4a where intensity in the 40 × 40 pixel array was integrated for each frame. A parallel cluster wavelet analysis (PCWA) algorithm reported in [34] was used to identify detected events apart from noise. This algorithm employs a continuous wavelet transform to efficiently identify signals amongst noise while providing intensity and Δ*t* (time domain FWHM) of each event. In this case, a ricker wavelet was used as it matches the waveform expected from events detected via single mode excitation. To determine the detected concentration, we use Equation (1), where *N* is the total number of events, *V_exc_* is the excitation volume determined by imaging the mode propagated in the waveguide and the width of the analyte channel, *T* is the total length of the time trace and Δ*t_avg_* is the average FWHM of detected events.

(1) $$c=\frac{NV_{exc}}{T\Delta t_{avg}},$$

In the total 120 s trace, 3036 fluorescent events were detected, yielding a detection rate of 25.3 events/second. The average FWHM of detected events was 0.567 ms. The excitation volume was found to be 4.3665 × 10^−10^ mL (mode width × mode height × channel width: 4.1 µm × 7.1 µm × 15 µm), determined by the FWHM of the mode and the depth of the channel. The detected concentration was determined to be 3.29 × 10^7^ beads/mL, or 54.62 fM. The initial proof-of-concept experiments that demonstrated sensing of 1 µm fluorescent beads using an on-chip DFB laser showed good agreement between the expected and experimental bead concentrations at femtomolar concentrations. The concentration demonstrated in [15] was 8 fM, thus illustrating a similar sensitivity of our device when compared with previous demonstrations using external excitation. The intensity histogram from the bead trace illustrates a tailed distribution (Figure 4b). This indicates that there are likely lower intensity signals that are missed. This could result from the bead traveling on the outer edges of the channel through a portion of the excitation volume with lower power. Despite this, the agreement of the expected concentrations indicates that the effect of this is small. Velocity of the beads is calculated by dividing the excitation width by each Δ*t* value. The velocity distribution indicates that some of the events are capped by the frame rate of the camera collection. Since the max frame rate is 2000 frames per second, our time resolution is limited to 0.5 ms. This corresponds to a max detectable velocity of 0.82 cm/s. Since we do see that the majority of signals are at the max velocity, it is likely that the true average velocity is higher. This can be rectified in the future by using a single photon counter to provide high resolution in the time domain.

### 3.2. All-in-One Nucleic Acid Assay Detection

**Figure 5:**
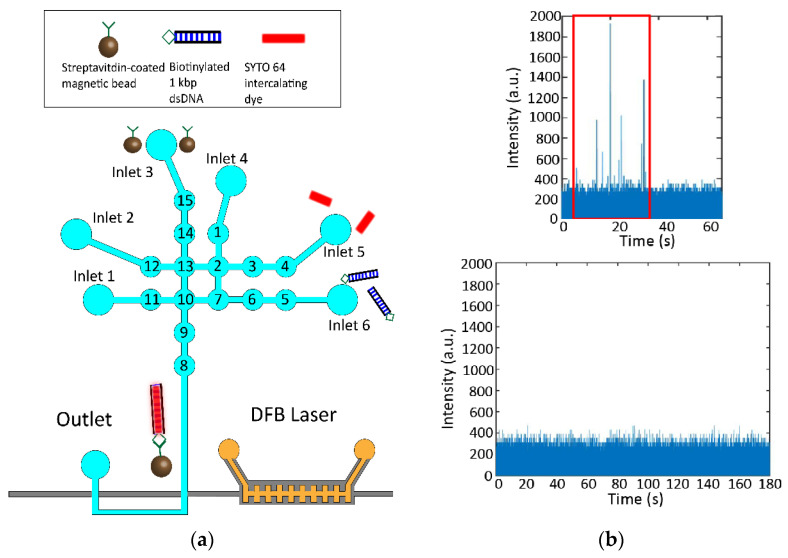
(**a**) Non-specific biomarker detection. Streptavidin-coated magnetic beads with capture probes are loaded into inlet 3 while the SYTO 64 intercalating dye and biotinylated 1kbp dsDNA are introduced into inlets 5 and 6, respectively. Inlets 1, 2, and 4 were loaded with 1xPBS buffer. Samples are brought together into the central mixing valves (13, 2, 7, 10) where they are mixed and incubated for the proper construct to be formed. Beads with captured fluorescently stained targets are pulled down with a magnet while excess targets and unconjugated probes are washed using buffer. The magnet is then removed, and the complete bead constructs are pushed to the detection region. (**b**) Fluorescence time domain trace illustrating peaks observed from fluorescently stained bead constructs (top). Control trace done in the absence of dsDNA targets illustrating no peaks above the noise floor (bottom).

The next step was to demonstrate the ability to perform a bioassay on our fully integrated lab-on-chip device via the nonspecific staining and detection of 1 kbp dsDNA (Figure 5a). 2 µL of 1 × 10^8^ magnetic beads per mL, 0.1 µM biotinylated 1 kbp dsDNA, and 50 µM SYTO 64 dye are loaded into three separate inlets. The 1 kbp dsDNA and the SYTO 64 dye are pushed into the central four mixing valves and incubated together for 10 min. After the dsDNA is stained with intercalating dye, the magnetic beads are added to the solution and incubated for a further 45 min. During the incubation, the DFB grating is filled with the gain medium and the pump beam is aligned in preparation. The completed assay was then cycled once through to the detection region of the device using sequential activation of valves 13–10–9–8.

From the nonspecific detection of stained dsDNA bead constructs, we observe an initial pulse of events followed by the rest of the trace illustrating no events (Figure 4b). This is the expected behavior since only one cycle was conducted. Due to a low number of signal counts, we noted that more cycles should be conducted in the specific nucleic acid detection to resuspend more bead constructs and maximize events. A control was carried out where the assay was conducted in the absence of the 1 kbp dsDNA, demonstrating no false positive events.

**Figure 6:**
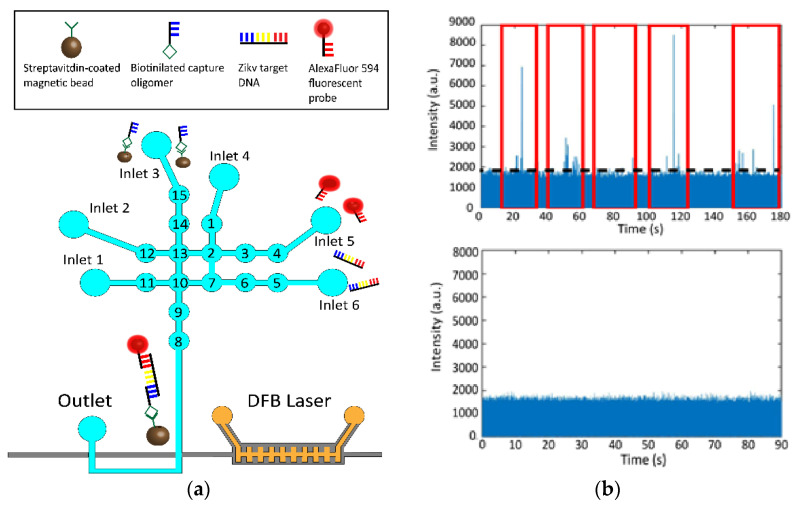
(**a**) Specific Zika assay detection. Magnetic beads with capture probes are loaded into inlet 3 while the AlexaFluor 594 fluorescent probes and Zika target DNA are introduced into inlets 5 and 6, respectively. Samples are brought together into the central mixing valves (13, 2, 7, 10) where they are mixed, heated and incubated for the proper construct to be formed. Beads with captured targets and fluorescent probes are pulled down with a magnet while excess targets and unconjugated probes are washed using buffer. The magnet is then removed, and the complete bead constructs are pushed to the detection region. (**b**) Fluorescence time domain trace illustrating peaks observed from fluorescently tagged bead constructs (top). Control trace done in the absence of Zika targets illustrating no peaks above the noise floor (bottom).

The final step was to complete a specific Zika bioassay from sample preparation to optical detection. Zika nucleic acids were optically detected in a magnetic bead-based assay that followed a similar protocol as described in [15] (Figure 6a). Initially, 2 µL of 1 × 10^9^ magnetic beads per mL, 5 µM Zika target, and 5 µM AlexaFluor probe were placed on three separate inlets and the first valves in each line are loaded. The remaining three inlets were loaded with 1xTE buffer to be used for cycling and washing steps. The three constituents are then brought together in the central four mixing valves and cycled to mix sample solution. The entire device was then placed on a thermoelectric heater that heated the chip to 40 °C for 5 min. The chip was left to incubate for a further 25 min before being returned to its original mount where the external pump beam is aligned to the DFB grating to enable to on-chip excitation. A magnet was then placed underneath the chip to pull down the magnetic bead constructs that now have target and fluorescent probes attached, and 1xTE buffer was used to rinse away excess target and fluorescent probes three times.

After being properly rinsed, the magnet was removed, and the buffer solution was mixed once to resuspend the bead constructs. Finally, the completed assay was pushed sequentially through to the detection region over the course of five cycles using 1xTE buffer to maximize the number of beads detected. The resulting time domain fluorescence trace is shown in Figure 6b.

**Figure 7:**
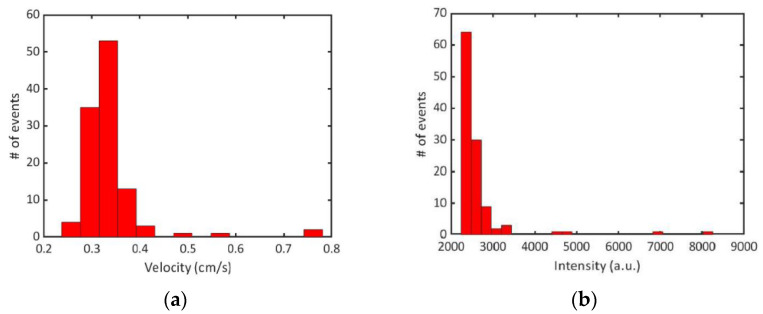
(**a**) Velocity distribution of detected events. Average velocity is 0.34 cm/s with a standard deviation of 0.07 cm/s. (**b**) Intensity distribution of detected events. Average intensity is 2055 with a standard deviation of 491.

The average velocity of the detected particles was 0.34 cm/s, which is notably lower than that of the constant flow experiments (Figure 7a). This can be explained by the fact that the velocity of these bead constructs relies on the flow of fluid generated by compressing the pneumatic valves rather than a negative pressure at the analyte channel outlet. A control was conducted where the assay was run identically, but in the absence of the Zika target ssDNA and illustrated no false positive events (Figure 6b).

## 4. Discussion

We have demonstrated the integration of an on-chip DFB laser with both optical detection and sample preparation functionalities on a PDMS lab-on-chip biosensor, which represents an important step towards the development of point-of-care lab-on-chip systems with unique advantages in lowering the spread of viral outbreaks by providing sensitive and rapid diagnoses. By using lab-on-chip optofluidic devices, we can minimize the necessary sample volumes and avoid risks of contamination and sample mishandling by conducting necessary assay preparation on the same chip. Furthermore, we can avoid inconsistent and tedious alignment of external excitation sources using an on-chip optofluidic DFB laser that is seamlessly integrated into the device design. The functionality of our device was demonstrated by the sensing of Zika nucleic acid on a magnetic bead assay, combining light source, sample preparation, and analysis on a single device. Future steps towards advancing point-of-care utility include replacing the camera sensor with a single photon detector [15] and using long-lasting surface modification of the fluidic channels [35] to further improve the sensitivity and efficiency down to previously demonstrated single molecule sensitivities. Additionally, the PCWA algorithm can be implemented in real-time to provide live results and further decrease sample-to-answer times [34]. This work provides concrete progress in the development of a rapidly deployable biosensor that has the potential to aid in reducing the spread of future viral outbreaks.

## Data Availability

Data available on request. The data presented in this study are available on request from the corresponding author.
